# Is It Inherited Paralysis?

**Published:** 1880-05-20

**Authors:** L. W. Weathers

**Affiliations:** Arkansas


### *IS IT INHERITED PARALYSIS?*

BY L. W. WEATHERS, M.D., OF ARKANSAS.

*Editor Southern Medical Record :*—You will please allow me space in your columns to present to our Profession the following case.

Mr. R——, by occupation a well-to-do farmer, stout, healthy, jovial gentleman, says he enjoys perfect health, and has all his life—and has every appearance of it—and while he is a good friend of the doctors when his family is sick, yet he laughs at them and tells them he has no need for their medicines.

Now the nature of the case to be considered. Mr. R. is now living with his third wife, who is the mother of five children—four boys and one girl; and, strange to say, while the children are as sprightly, fine looking as you generally find, the boys all become paralyzed and die at the age of two and four years. Their intellectual development is rather precocious.

The mother is a stout, healthy lady and from healthy parentage, a remarkably stout, healthy family, for I am personally acquainted with her people. The difference in age of wife and husband is, I presume, twenty years, he being her senior.

I know that paralysis is said to be traceable to vice, intemperance, etc., but we can find nothing of this nature to give us any light. On both sides of the house they are perfectly healthy. Mr. R—— is the father of seven children, and at present his girls are all living, and no symptoms of this disease with them. Even the girl by his present or third wife is perfectly healthy, and being physician to the family I affirm that what I have herein stated is true.

Having studied this to the best advantage and finding no discernible cause, I respectfully submit it to the Profession for further information.

[We hope that some member of the Profession will respond to the above communication.—ED. RECORD.]

